# Characterisation of ovine lymphatic vessels in fresh specimens

**DOI:** 10.1371/journal.pone.0209414

**Published:** 2019-01-16

**Authors:** Hung-Hsun Yen, Christina M. Murray, Elizabeth A. Washington, Wayne G. Kimpton, Helen M. S. Davies

**Affiliations:** 1 Melbourne Veterinary School, The University of Melbourne, Parkville, Victoria, Australia; 2 Research Center for Animal Biologics, National Pingtung University of Science and Technology, Neipu, Taiwan; Tokat Gaziosmanpasa University, TURKEY

## Abstract

**Background and aim:**

The development and use of experimental models using lymphatic cannulation techniques have been hampered by the lack of high-quality colour imaging of lymphatic vessels in situ. Most descriptions of lymphatic anatomy in sheep have historically depended on schematic diagrams due to limitations in the ability to publish colour images of the lymphatic vessels with decent resolution. The aim of this work was to encourage more widespread use of the ovine cannulation model by providing clear photographic images identifying the location and anatomical layout of some major lymphatic ducts and their in situ relationship to surrounding tissues.

**Methods:**

The cadavers of the sheep were collected after they had been euthanized at the end of animal trials not associated with this study. The lymphatics were dissected and exposed to show their appearance in the surrounding tissues and their relationship to other organs. Patent Blue was used to locate lymphatic vessels in exploratory preparations. However, in order to present the natural appearance of the vessels, we used minimal dissection and dye was not used for the photographed examples. Instead, we have indicated the course of the vessels with lines where their position is less clear.

**Results and conclusion:**

In this paper, we have used sheep specimens as examples to show characteristic images of lymphatic vessels. The images of in situ lymphatics and lymph nodes combined with schematic summaries provide a concise illustration of the lymphatic drainage scheme in sheep.

## Introduction

Lymph carries key physiological and immunological factors, such as cytokines and leukocytes, to and from the local lymph nodes for the induction and propagation of immune responses. In addition, it provides the opportunity to detect the presence in the lymph of antigens that are produced by pathogens or expressed by tumor cells, and allows real-time sampling during the course of an immune response. Consequently, direct access to lymph can generate useful information regarding diseases and immunity at these drainage sites.

One successful application of lymphology has been the establishment of lymphatic cannulation models to access lymph draining specific target organs in large animal models such as sheep [1]. In this example, knowledge of the morphology of the lymphatic vessel and its drainage is essential. Sheep have anatomical and physiological structures that are similar to those in humans and thus are ideal models for biomedical studies [2]. Indeed, the lymphatic cannulation models in sheep have been broadly applied to many studies, such as in immune cells circulation [3], assessing the immune responses following flu vaccine intra-nasal delivery [4] and innate immunity following vaccination with poly(I:C)-containing liposomes [5].

**Fig 1:**
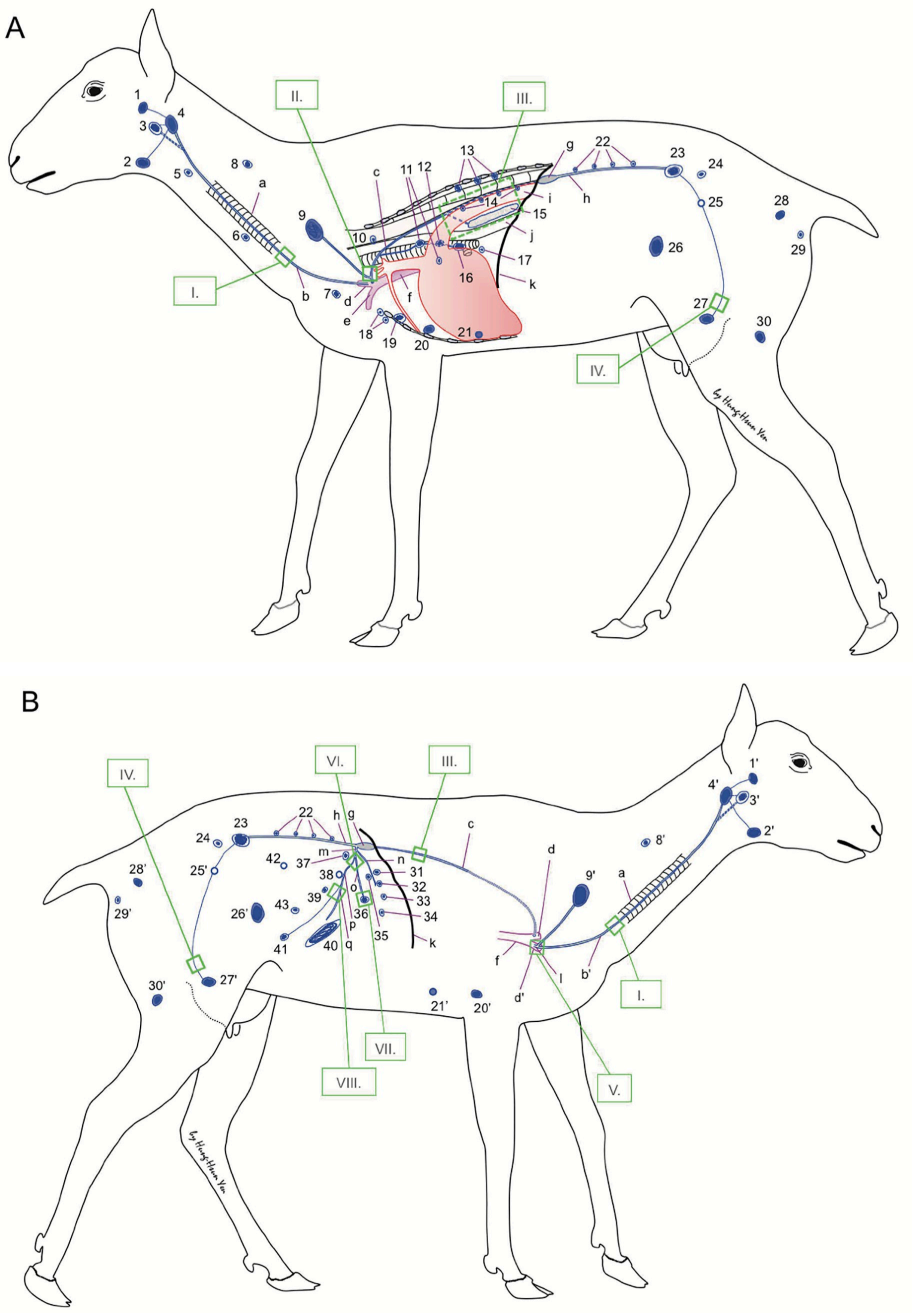
Survey diagram of the lymph nodes of sheep. Two schematic images illustrate the principal lymphatic vessels and the lymph nodes of sheep. **I.** Images showing the tracheal trunks are available in the previous report by Yen at al (Yen et al., 2006) [7]. **II.** The image showing the junction of the thoracic duct and the efferent lymphatic of the superficial cervical lymph node(s) on the left can be found in the previous report by Yen at al (Yen et al., 2009) [8]. **III.** A segment of the thoracic duct dorsal to the aorta and the caudal mediastinal lymph node in the caudal thoracic cavity on the right are illustrated in Fig 2. **IV.** Images showing the efferent lymphatic vessels of the mammary glands are available in the previous reference by Yen et al (Yen et al., 2016) [9]. **V.** The right lymphatic duct, the tracheal trunk and the efferent lymphatic of the superficial cervical lymph node(s) on the right are depicted in Fig 3. **VI.** The visceral, hepatic and intestinal trunks are depicted in Fig 4. **VII.** The afferent and efferent lymphatic vessels of a hepatic lymph node are depicted in Fig 5. **VIII.** The efferent lymphatic of the ileocolic lymph nodes and the jejunal trunk are shown in Fig 6. 1&1’: left and right parotid lymph nodes; 2&2’: left and right mandibular lymph nodes; 3&3’: left and right medial retropharyngeal lymph nodes; 4&4’: left and right lateral retropharyngeal lymph nodes; 5: cranial deep cervical lymph nodes; 6: middle deep cervical lymph nodes; 7: caudal deep cervical lymph nodes; 8&8’: left and right accessary superficial cervical lymph nodes; 9&9’: left and right superficial cervical lymph nodes; 10: costocervical lymph nodes; 11: cranial mediastinal lymph node(s); 12: middle mediastinal lymph node(s); 13: intercostal lymph nodes; 14: thoracic aortic lymph nodes; 15: caudal mediastinal lymph nodes; 16: tracheobronchial lymph center; 17: pulmonary lymph nodes (left and right); 18: cranial and caudal sternal lymph nodes; 19: costoaxillary lymph nodes; 20&20’: left and right axillary lymph nodes; 21&21’: left and right accessary axillary lymph nodes (inconstant); 22: lumbar aortic lymph nodes; 23: medial iliac lymph node(s); 24: sacral lymph nodes; 25&25’: left and right iliofemoral lymph nodes (inconstant); 26&26’: left and right subiliac (or prefemoral) lymph nodes; 27&27’: left and right mammary (or superficial inguinal) lymph nodes; 28&28’: left and right sciatic (or ischiadic) lymph nodes; 29&29’: left and right anorectal lymph nodes; 30: left and right popliteal lymph nodes; 31: atrial lymph nodes; 32: reticular lymph nodes; 33: omasal lymph nodes; 34: dorsal abomasal lymph nodes; 35: right ruminal lymph nodes; 36: hepatic lymph nodes; 37: renal lymph nodes; 38: celiac and cranial mesenteric lymph nodes (inconstant); 39: pancreaticoduodenal lymph nodes; 40: jejunal lymph nodes; 41: ileocolic lymph nodes; 42: colic nodes; 43: caudal mesenteric lymph nodes (inconstant); a: trachea; b&b’: left and right tracheal trunks; c: thoracic duct; d&d’: left and right external jugular veins; e: left subclavian vein; f: cranial vena cava; g: cisterna chyli; h: lumbar trunk; i: aorta; j: esophagus; k: diaphragm; l: right lymphatic duct; m: visceral trunk; n: gastric trunk; o: hepatic trunk; p: intestinal trunk; q: jejunal trunk.

The most practical approach to learning the anatomy of lymphatic vessels, without undertaking dissections with an expert, is through the use of photographic images. However, in most papers describing lymphatic drainage and lymphatic cannulation techniques, only schematic images are available due to historical limitations on publishing clear colour photographs with acceptable resolution. An additional complication for human lymphatic anatomy is the difficulty in accessing dissection material. Fresh cadavers of animal species such as sheep, in which surgical procedures for catheterizing the lymphatic vessels have been well investigated and established, present an alternative model [6–9]. Indeed, images showing the cisterna chyli together with the thoracic duct, the point of entry of the thoracic duct into the external jugular vein and the tracheal trunk on the left in pigs have been published [10, 11]. The injection of rubber compounds has been used to show the lymphatics in relief, and while this gives a dramatic three-dimensional visualization, it does not show the vessels in their natural state [12]. Photographic images of the tracheal trunk [7], the junction of the thoracic duct and an efferent lymphatic vessel of the superficial cervical lymph nodes [8] and the mammary lymphatic vessels [9] in sheep are also available. However, photographic images showing other major lymphatic vessels are either not available, or are black and white images [13, 14] even though the methods for catheterizing these lymphatics have been reported [6, 15, 16]. In this study, we took sheep as an example to produce images of major lymphatic vessels in fresh sheep cadavers, including afferent and efferent hepatic ducts, jejunal and ileocolic lymphatics, the major visceral, hepatic and intestinal trunks, the thoracic duct in the caudal thorax, and the right lymphatic duct where lymph rejoins the blood circulation. The locations of these are summarized in the schematic diagram of the key lymphatic vessels and the lymph nodes of sheep (Fig 1).

## Materials and methods

The cadavers of the sheep were collected after they had been euthanised at the end of animal trials (AEEC No. 1212422.3, 1312857.2, 0704987, 0706727 and 0911109.4) not associated with this study. Those trials were conducted at the Faculty of Veterinary and Agricultural Sciences (FVAS) animal facility and were carried out with the permission of the Melbourne University Animal Ethics Committee. The lymphatics were dissected and exposed to show their appearance and relationship to the surrounding tissues. A number of sheep (4–5 for each image) were initially dissected to establish the best surgical approach to locate and clearly demonstrate the lymphatics. Sheep that are presented in the photographs presented did not have patent blue dye and were presented where possible with minimal dissection. The video that shows the jejunal trunk and the efferent lymphatic of the ileocolic lymph nodes was obtained from the animals of a recent trial (AEEC No. 1413429; Prof Robin Gasser kindly provided us with the animals’ cadavers). Images were taken using a Nikon camera (D200) and an iPhone SE and Microsoft PowerPoint 2011 was used for image and text editing.

## Results

Based on the description in the previous references [17, 18], we constructed a schematic survey diagram to illustrate the constant lymph nodes and the major lymphatic vessels in sheep (Fig 1). Several inconstant lymph nodes that may have clinical significance, such as the accessory axillary lymph nodes, are also depicted in this figure. However, detailed information about the individual variations of inconstant lymph nodes in sheep and goats is not included in this figure but can be found in Tanudimadja’s thesis [18].

Lymph from lymph nodes in the head and neck on the left, the thorax and any region caudal to the diaphragm drains to the thoracic duct (c, left lymphatic duct, Fig 1A and 1B), whereas the right lymphatic duct collects lymph from lymph nodes in the head, neck and cranial thorax on the right side (Fig 1B). The right lymphatic duct and the thoracic duct join the jugular veins near the confluence of the external jugular vein and the cranial vena cava on each side. The tracheal trunk (b, Fig 1A) gathering lymph from the left side of the head and neck may terminate in the thoracic duct or enter the external jugular vein, adjacent to its confluence with the subclavian vein on the left. Lymph from the gastric trunk, hepatic trunk and the intestinal trunk all drains into the visceral trunk and then enters the lumbar trunk or the cisterna chyli (g) in the abdomen (Fig 1).

### Ovine thoracic duct and right lymphatic duct

As depicted in Fig 1, the thoracic duct (left lymphatic duct—c) collects lymph draining the regions caudal to the diaphragm including the hindlimbs, gastrointestinal tracts and other abdominal organs. The popliteal (30), anorectal (29), sciatic (or ischiadic—28), sacral (24), subiliac (or prefemoral—26), mammary (in female) or superficial inguinal (in male) (27) and the inconstant iliofemoral lymph nodes (25) all drain their lymph into the medial iliac lymph center (23). Lymph from the medial iliac lymph center drains into the lumbar trunk (h).

**Fig 2:**
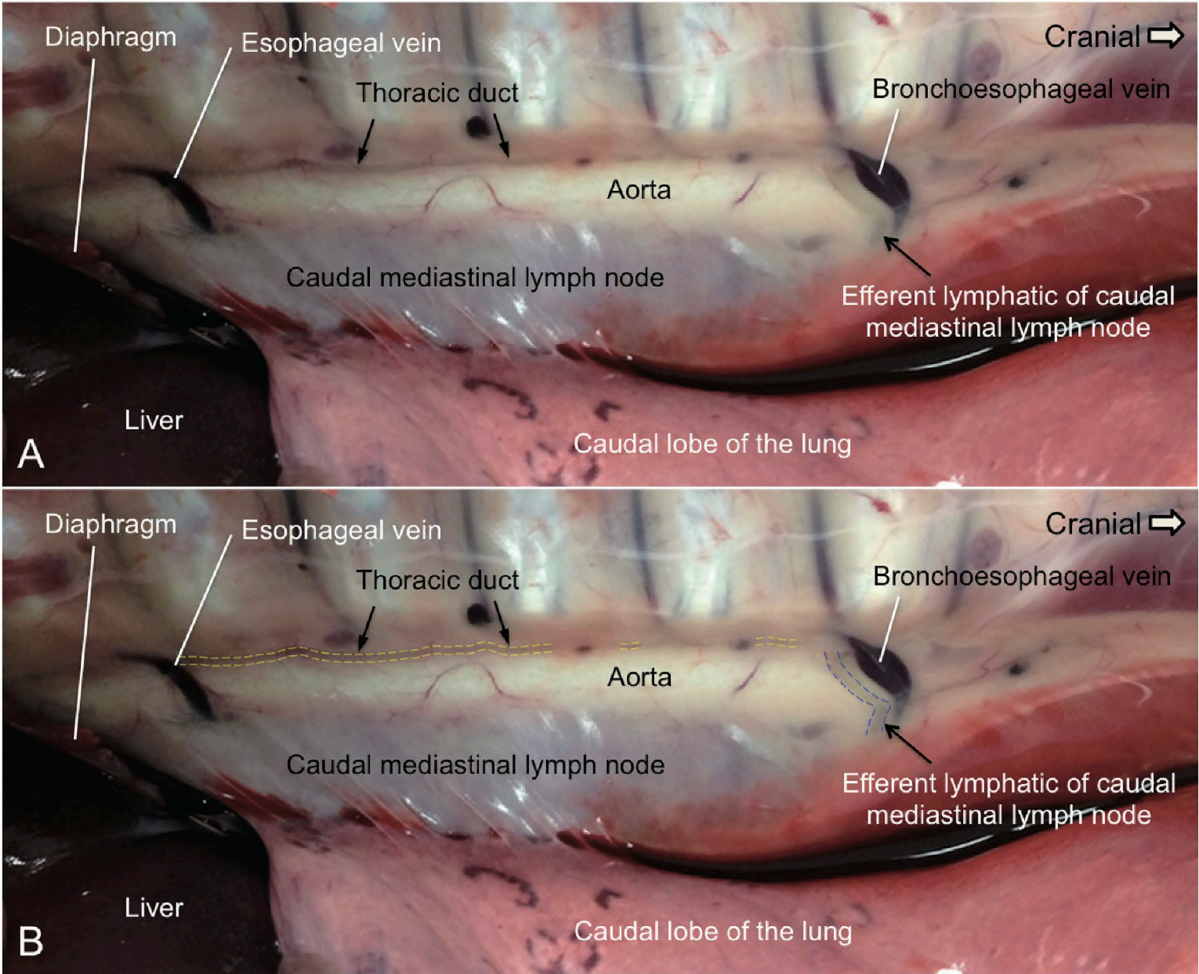
The dissection of an ovine thoracic duct in the caudal thorax on the right. A. The locations of the thoracic duct, the caudal mediastinal lymph node and a short segment of its efferent lymphatic on the right side of the thorax are depicted in this image. The thoracic duct was located dorsal to the aorta right after it passed through the diaphragm and entered the thoracic cavity. In most animals, the thoracic duct is to some extent shifted to the right in the caudal thorax. An efferent lymphatic vessel of the caudal mediastinal lymph node embedded in fat, departed at the cranial end of the lymph node and led dorsally to the thoracic duct. The setting for the contrast and brightness of this image is for the presentation of the lymphatics rather than the liver and the diaphragm. The depth differences between the thoracic duct and the livers in this image are huge; therefore the liver and the diaphragm look dark. This figure is positioned with cranial to the right. B. The photograph is the same as Fig 2A. In this figure, the yellow dotted lines indicate the location of three segments of the thoracic duct. These segments are not embedded in the adipose tissues. The blue dotted lines mark the visible part of the efferent lymphatic vessel of the caudal mediastinal lymph node.

The cisterna chyli (g), an enlarged segment of the lumbar lymphatic that is located caudal to the diaphragm, resembles a “pool”. Theoretically, it provides a buffer compartment assisting the adjustment of lymph flow that corresponds to the changes of pressure in different respiratory patterns. The cisterna chyli continues as the thoracic duct as it enters the thoracic cavity. The thoracic duct is located dorsal to the aorta right after its entry into the thoracic cavity (Fig 2). In most sheep, the thoracic duct is somewhat shifted to the right in the caudal thorax [17, 19]. However, the thoracic duct in the caudal thorax can be rationally approached from the left hand side in pigs [10].

The principal caudal mediastinal lymph node in the sheep is a long prominent lymph node located between the caudal lobes of the lung and ventral to the aorta in the caudal thoracic cavity (Fig 1A—15 and Fig 2). A short segment of the efferent lymphatic emerging from the cranial pole of the caudal mediastinal lymph node and coursing across the right lateral surface of the aorta to the thoracic duct is shown in this photograph and depicted as a dotted line in Fig 1A–Box III. Multiple efferent lymphatics from different portions of the caudal mediastinal lymph node with variations in the courses of their ducts to the thoracic duct have been observed previously [20].

Lymph from the major lymph nodes, including the tracheobronchial lymph center (Fig 1A—16) and the caudal mediastinal and cranial mediastinal lymph nodes (15, 11) draining the lungs and the heart, streams into the thoracic duct. The thoracic duct terminates in the external jugular vein (d) near the confluence of the external jugular and subclavian veins (e) on the left (Fig 1A–Box II). Previous findings [8, 19] indicate that the efferent lymphatic of the superficial cervical lymph node usually merges with the thoracic duct adjacent to the point of entry of the thoracic duct into the external jugular vein on the left. The left tracheal trunk(s) (b) that originate from the lateral retropharyngeal lymph node(s) (4), and possibly the medial retropharyngeal lymph node(s) (3) in some animals, collect lymph from the head and the ventral cervical regions and also terminate in the external jugular vein near its junction with the thoracic duct on the left. The left tracheal trunk(s) may also coalesce with the thoracic duct.

**Fig 3:**
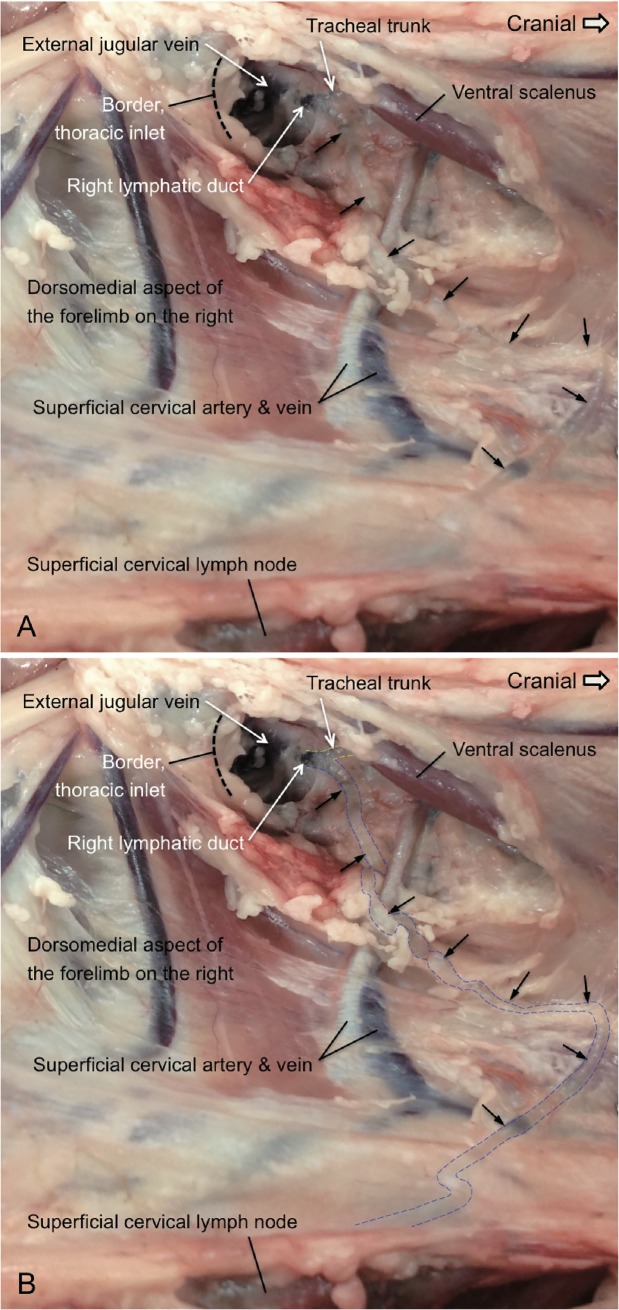
The dissection of an ovine right lymphatic duct. A. The locations of the right lymphatic duct at its point of entry into the external jugular vein on the right are depicted in this image. The efferent lymphatic vessel of the superficial cervical lymph node(s) on the right coursed medial to the superficial cervical artery and vein toward the thoracic inlet. This lymphatic merged with the tracheal trunk at the location just cranial to the thoracic inlet to become the right lymphatic duct. The black arrows depict the efferent lymphatic vessel of the superficial cervical lymph node(s). This figure is positioned with cranial to the right and shows the dorsal aspect of the caudal cervical and axillary regions medial to the forelimb on the right. B: This photograph is the same as Fig 3A. In this image, the yellow dotted lines mark the location of the tracheal trunk and the blue dotted lines indicate the course of the efferent lymphatic of the superficial cervical lymph node. Ventral scalenus: the ventral scalenus muscle.

As illustrated in Fig 3, the efferent lymphatic of the superficial cervical lymph node (black arrows) merges with the right tracheal trunk to form the right lymphatic duct before its entry into the external jugular vein on the right (Fig 1B–Box V). The finding regarding the right lymphatic duct in this figure is compatible with a previous description [19]. Based on the location of the right duct in this figure, it is likely that the efferent lymphatic vessel of the cranial mediastinal lymph node(s) does not always enter the right duct. Previous results indicate that efferent lymphatic vessels of the cranial mediastinal lymph node(s) enter the thoracic duct in sheep, cattle and horses [8, 17], although different anatomical findings are reported in dogs [21]. These anatomical differences should be noted when studies of applying the lymphatic cannulation models for harvesting pulmonary lymph in dogs and sheep are conducted [1].

### Ovine visceral trunk, hepatic trunk and intestinal trunk

**Fig 4:**
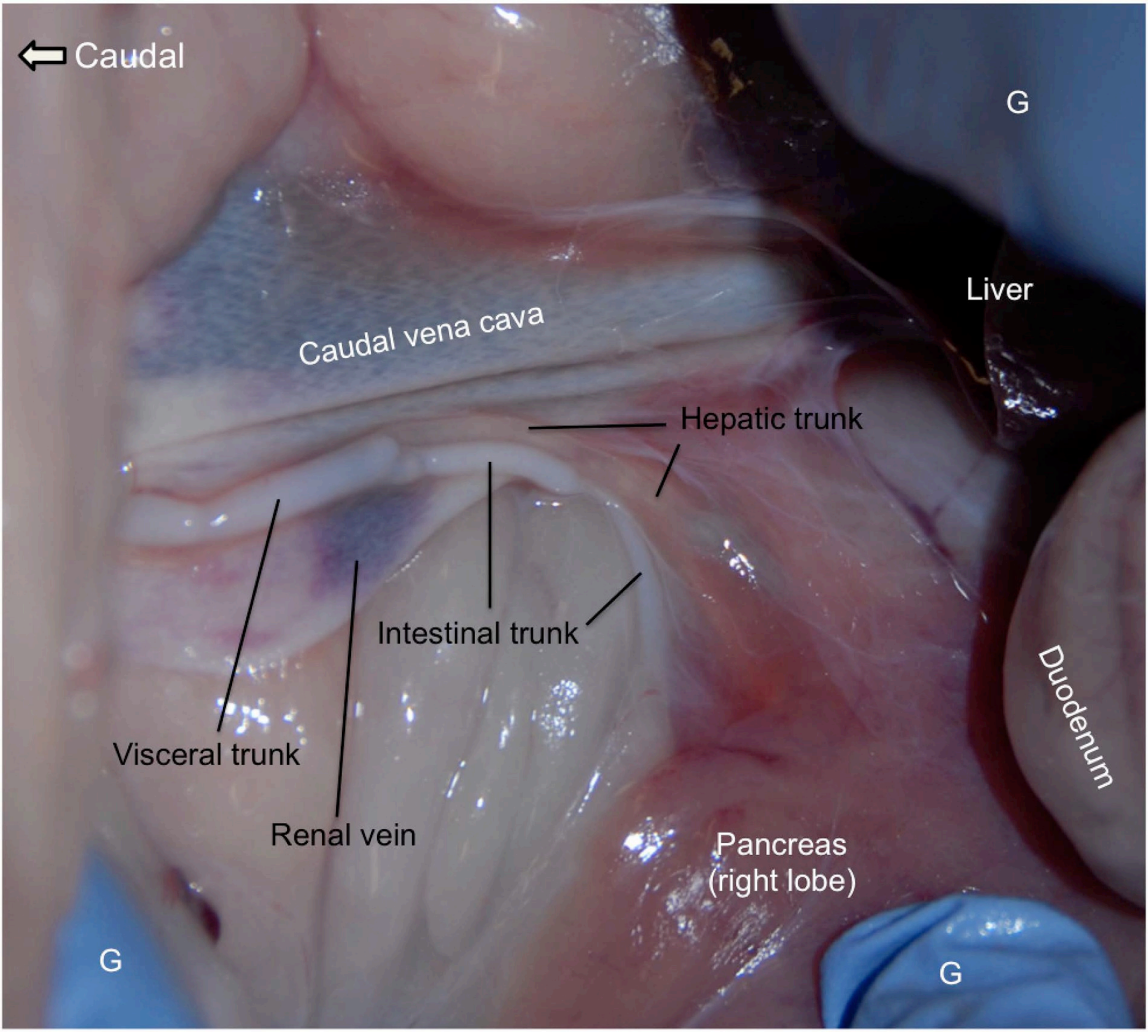
The dissection of an ovine visceral trunk, hepatic trunk and intestinal trunk. The locations of the visceral trunk and the confluence of the hepatic and intestinal trunks are present in this image. Lymph in the intestinal trunk contains abundant lipids following normal feeding and is shown by the cloudy appearance. Lymph from the intestinal trunk drains into the visceral trunk and thus the visceral trunk also presents the “milky” color. As depicted in Fig 1B, the visceral trunk collects lymph from many major organs in the abdominal cavity. Lymph from the gastric trunk, hepatic trunk and intestinal trunk all streams into the visceral trunk. The visceral trunk enters the lumbar trunk next to its junction with the cisterna chyli. This figure is positioned with cranial to the right and shows the ventrolateral aspect of the region caudal to the liver on the right. G: gloves.

The visceral trunk, which is the principal lymphatic collecting lymph from most of the organs in the abdomen, joins the lumbar trunk at its junction with the cisterna chyli (Fig 1B—m). Three main lymphatic vessels, the hepatic (o), intestinal (p) and gastric (n) trunks all enter the visceral trunk (Fig 1B). Fig 4 shows the hepatic trunk and the intestinal trunk emptying into the visceral trunk (Fig 1B–Box VI). The gastric trunk, which collects lymph draining the stomachs, is not shown in Fig 4. Lymph in the intestinal and visceral trunks has the “milky” appearance due to the presence of chylomicrons in their lumens, whereas the color of lymph in the hepatic trunk remains light yellowish. There are valves at the junction between the hepatic and visceral trunks that stop the backflow of “milky” lymph into the hepatic trunk.

### Hepatic lymphatics

**Fig 5:**
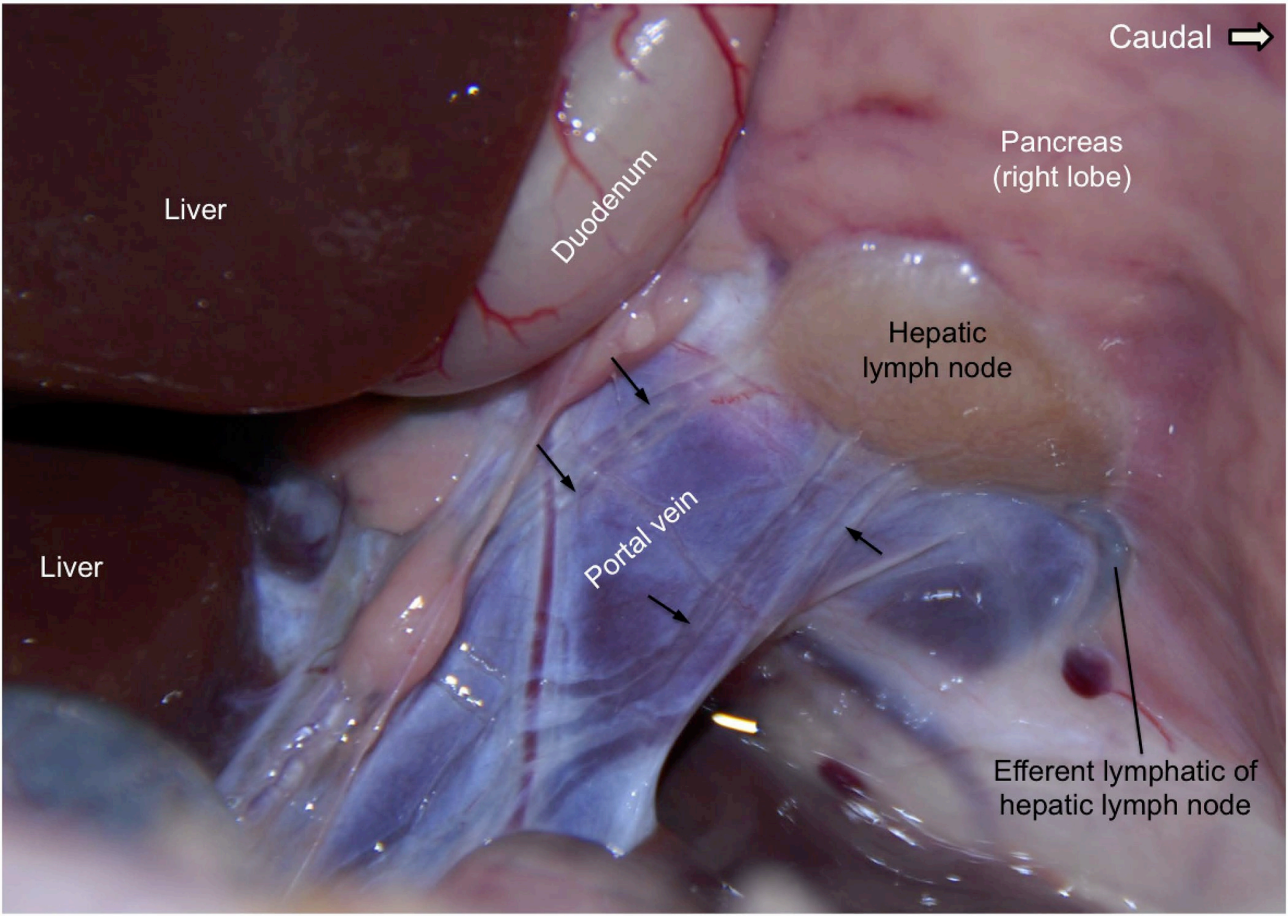
The dissection of one ovine hepatic lymph node and its afferent and efferent lymphatic vessels. The location of a hepatic lymph node at the junction of the portal vein and pancreas is shown in this image. Multiple afferent lymphatic vessels coming from the liver alongside the portal vein are entering this lymph node. There was an efferent lymphatic, a branch of the hepatic trunk, leaving this lymph node. This image demonstrates the usual number of afferent and efferent lymphatic vessels of a lymph node. This figure is positioned with cranial to the left and caudal to the right and shows the ventrolateral aspect of the tissues between the pancreas and the liver on the right hand side. Portal vein: the wall of the portal vein is shown lying beneath the clear afferent lymphatics.

The hepatic lymph node in Fig 5 represents a typical illustration for the number of afferent and efferent lymphatic vessels of a lymph node. Multiple afferent lymphatic vessels coming from the liver and running alongside the portal vein are shown entering this hepatic lymph node (small black arrows). Each afferent lymphatic vessel divides into smaller branches on the capsule of the lymph node or at the sub-capsular sinus (not labeled). Only one efferent lymphatic (labeled) leaves this hepatic lymph node and that lymphatic joins the hepatic trunk.

### Jejunal and ileocolic lymphatics

**Fig 6:**
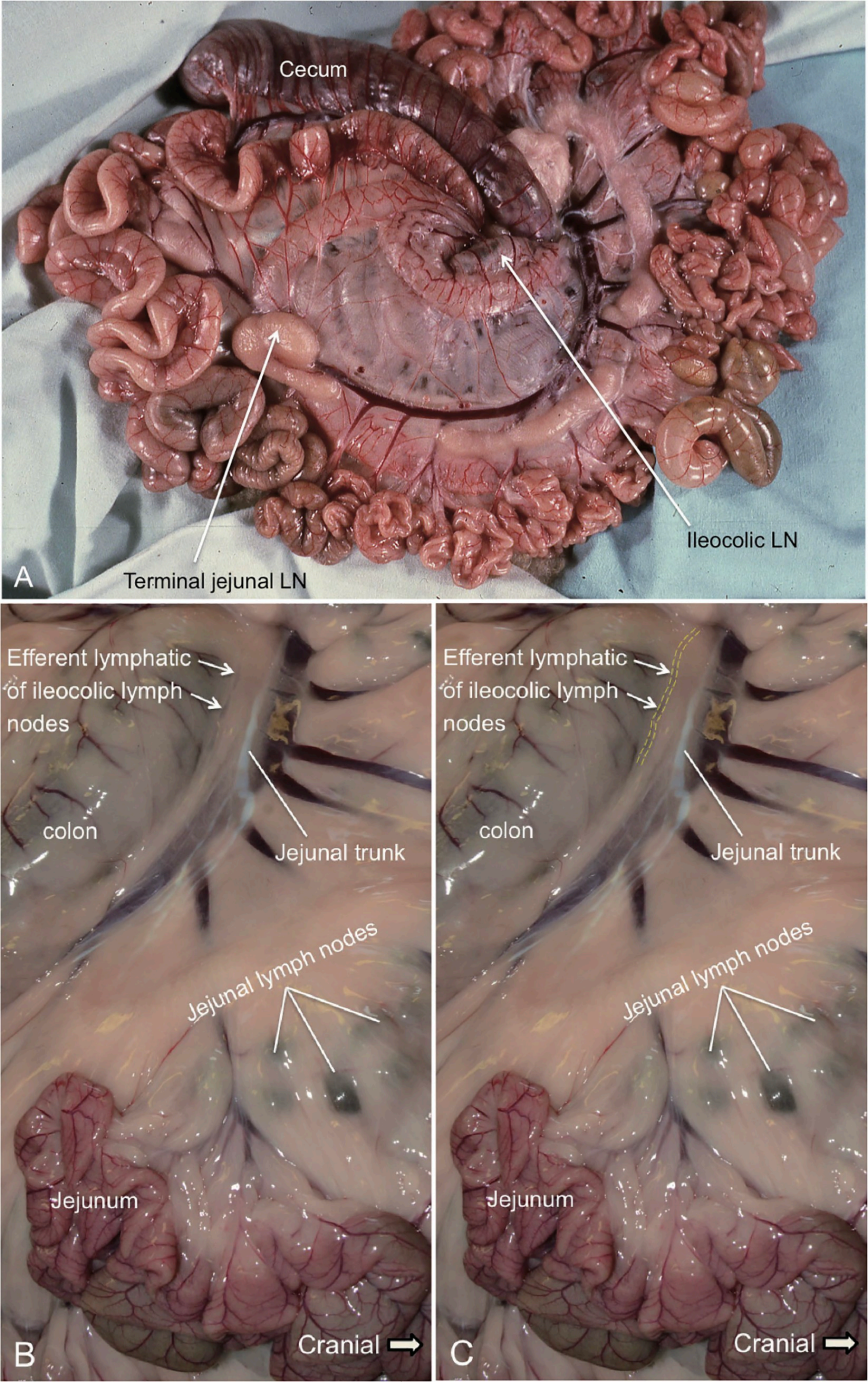
The dissection of an ovine jejunal lymphatic trunk and the efferent lymphatic of the ileocolic lymph node(s). A. The lymph nodes and lymphatics exposed in the initial dissection of ovine mesentery. The ileocolic lymph node is shown at the end of the cecum (cecocolic junction). The large, discrete terminal jejunal lymph node indicated in the image has previously been referred to as the ileocecal lymph node and drains the terminal ileum [13]. B. The locations of the jejunal lymphatic trunk which is a branch of the intestinal trunk, and an efferent lymphatic of the ileocolic lymph node are depicted in this image. This efferent lymphatic of the ileocolic lymph node(s) is a segment of the lymphatic that is located proximal to its join to the intestinal trunk. The jejunal lymphatic shows the usual “milky” appearance due to the presence of chyle in its lumen. The jejunal trunk collects lymph draining the jejunal lymph nodes. The jejunal trunk is associated with the mesentery that lies on the colon. This figure is positioned with cranial to the right. C: This photograph is the same as Fig 6B. In this image, the yellow dotted line marks a segment of the efferent lymphatic of the ileocolic lymph nodes. The efferent lymphatic of the ileocolic lymph node and the jejunal lymphatic trunk are also presented in the video (S1 File) attached.

The locations of the jejunal lymphatic trunk and an efferent lymphatic of the ileocolic lymph node are depicted in Fig 6. As illustrated in Fig 1B (Box VIII), the portion of the efferent lymphatic of the ileocolic lymph node(s) that is shown in Fig 6B and 6C is a segment of the lymphatic that is located proximal to its point of entry into the intestinal trunk. The jejunal and ileocolic lymph nodes are indicated in Fig 6A. The efferent lymphatic of the ileocolic lymph node(s) and the jejunal trunk that collects lymph draining the jejunal lymph nodes both enter the intestinal trunk. The jejunal lymphatic shows the usual “milky” appearance due to the presence of chyle in its lumen, whereas the efferent lymphatic of the ileocolic lymph node(s) does not. This common finding indicates that absorption of chylomicrons (a major constituent of chyle) predominantly occurs from the jejunum and not from the caudal segment of the small intestine. Previously it has been demonstrated that the levels of lipids (triglyceride, which is an essential component of chylomicrons) corresponded to the milky appearances of the lymph samples [11].

## Conclusions

In this article, we have described the anatomy of some major lymphatic ducts using photographic images of the dissected tissues to show both their appearance in fresh specimens and their anatomical relationship to surrounding tissues. Previous descriptions of these tissues have relied on schematic diagrams and the work reported here provides a visual compendium to allow these important lymphatic vessels to be found and identified reliably in fresh specimens. We have also highlighted variations in thoracic duct and right lymphatic duct anatomy, which are important in understanding the relationship between the lymphatic and blood circulatory systems.

## Supporting information

S1 File This is the video showing the efferent lymphatic of the ileocolic lymph node and the jejunal lymphatic trunk. (MP4)
